# Computer Programs and Methodologies for the Simulation of DNA Sequence Data with Recombination

**DOI:** 10.3389/fgene.2013.00009

**Published:** 2013-02-01

**Authors:** Miguel Arenas

**Affiliations:** ^1^Centre for Molecular Biology “Severo Ochoa,” Consejo Superior de Investigaciones CientíficasMadrid, Spain

**Keywords:** simulation, recombination, recombination breakpoints, recombination hotspots, DNA sequences, recombination phylogenetic bias

## Abstract

Computer simulations are useful in evolutionary biology for hypothesis testing, to verify analytical methods, to analyze interactions among evolutionary processes, and to estimate evolutionary parameters. In particular, the simulation of DNA sequences with recombination may help in understanding the role of recombination in diverse evolutionary questions, such as the genome structure. Consequently, plenty of computer simulators have been developed to simulate DNA sequence data with recombination. However, the choice of an appropriate tool, among all currently available simulators, is critical if recombination simulations are to be biologically meaningful. This review provides a practical survival guide to commonly used computer programs and methodologies for the simulation of coding and non-coding DNA sequences with recombination. It may help in the correct design of computer simulation experiments of recombination. In addition, the study includes a review of simulation studies investigating the impact of ignoring recombination when performing various evolutionary analyses, such as phylogenetic tree and ancestral sequence reconstructions. Alternative analytical methodologies accounting for recombination are also reviewed.

## Introduction

Recombination constitutes a basic and dominant mechanism in molecular evolution, increasing genetic diversity before natural selection operates on the new sequence. Recombination is widespread across nuclear genomes (e.g., Awadalla, 2003; Tsaousis et al., 2005; Fraser et al., 2007; Gaut et al., 2007; Duret and Arndt, 2008) and the importance of its understanding has been long recognized, with crucial implications for genome structure (Reich et al., 2001), phenotypic diversity (Zhang et al., 2002), and genetic diseases (Daly et al., 2001). Moreover, ignoring recombination may bias phylogenetic reconstructions (e.g., Posada, 2001; Posada and Crandall, 2002; Beiko et al., 2008), and the derived inferences (e.g., Schierup and Hein, 2000a; Feil et al., 2001; Anisimova et al., 2003; Arenas and Posada, 2010a,b,c).

The evolutionary importance of recombination (e.g., Robertson et al., 1995; Lukashev, 2005) calls for its accurate detection and measurement (see, Martin et al., 2011). Although some analytical methods have shown an overall better performance than others (Posada and Crandall, 2001; Wiuf et al., 2001), the choice of an appropriate tool also depends on the particular analysis (e.g., detection of recombination breakpoints or estimation of recombination rates), computational costs (some methods are computationally expensive), and the genetic marker. I recommend the following two reviews for helping users to make choices for appropriate methods and computer tools for recombination inference (Posada et al., 2002; Martin et al., 2011).

Computer simulations aim to mimic real world processes. They allow the study of mechanisms that may alter processes or the understanding of complex systems that are analytically intractable (Peck, 2004). Indeed, the simulation of evolutionary histories is commonly used for hypothesis testing (e.g., Arenas et al., 2008; Pierron et al., 2011), to verify and compare analytical methods or programs (e.g., Westesson and Holmes, 2009; Marttinen et al., 2012), to analyze interactions among evolutionary processes (e.g., Arenas et al., 2012, 2013), or to estimate evolutionary parameters (e.g., Wilson et al., 2009; Beaumont, 2010). Importantly, the choice of an appropriate simulator is critical because simulations should be as realistic as possible in order to mimic a given biological scenario. Although several studies have already reviewed computer simulators in population genetics from global perspectives (e.g., Liu et al., 2008; Arenas, 2012; Arenas and Posada, 2012; Hoban et al., 2012), they have not explored particular methodologies for the simulation of DNA sequences with recombination.

The present study provides an overview of the capabilities of available computer tools and methodologies, and suggests recommendations, for the simulation of DNA sequences with recombination. It also describes some applications of simulated datasets with recombination to show the importance of including recombination in evolutionary analyses. Alternative analytical methodologies that consider recombination are also suggested.

## Computer Programs for the Simulation of DNA Data Under Recombination

Recombination can be simulated by the two major simulation approaches commonly used in population genetics, the forward in time (forward-time, where the evolutionary history of an entire population is simulated from the past to the present; e.g., Epperson et al., 2010) and the coalescent (backward-time, which describes a backward in time genealogical process from a sample of genes to a single ancestral copy; e.g., Nordborg, 2007; Wakeley, 2008). The forward-time approach can simulate complex processes, including gene–gene interactions and complex selection (e.g., Calafell et al., 2001; Peng et al., 2007), but coalescent simulations are computationally faster and can be recommended for extensive simulation studies (e.g., Beaumont et al., 2002). Table 1 shows an overview of currently available computer programs, for both coalescent and forward-time approaches, to simulate DNA sequences with recombination.

**Table 1:** **Commonly used software for direct simulation of DNA sequences under recombination**.

Program	Evolutionary history	Recombination algorithm	Recombination hotspots	Other evolutionary processes	Substitution model	Rate variation	Intracodon recombination	Indels	OS	Citation
CodonRecSim	Coalescent	SCR	No	No	Cod^b^: GY94	No	No	No	SC, Win	Anisimova et al. (2003)
Recodon/NetRecodon	Coalescent	SCR^a^	No	D, Pm	Nt: All; Cod^b^: GY94	G, I	Yes (NetRecodon)	No	All	Arenas and Posada (2007, 2010a)
SIMCOAL2	Coalescent	SCR	Yes	D, Pm	Nt: JC, K2P	No	No	No	Linux, Win	Laval and Excoffier (2004)
Fastsimcoal	Coalescent	SMC	Yes	D, Pm	Nt: JC, K2P	No	No	No	Linux, Mac, Win	Excoffier and Foll (2011)
Mlcoalsim	Coalescent	SCR	Yes	D, Pm	Nt: JC, K2P	G, I	No	No	All	Ramos-Onsins and Mitchell-Olds (2007)
TREEEVOLVE	Coalescent	SCR	No	D, Pm	Nt: All	G	No	No	SC, Mac	Grassly and Rambaut (1997)
SPLATCHE2	Forward, coalescent	SCR	No	D, Pm	Nt: JC, K2P	No	No	No	Linux, Win	Ray et al. (2010)
GenomePop	Forward	CO	Yes	D, Pm, S	Nt: JC, GTR; Cod: MG94	No	Yes	No	SC, Linux, Win	Carvajal-Rodriguez (2008)
SFS_CODE	Forward	CO, SB	Yes	D, Pm, S	Nt: All; Cod: Nt^c^	G	No	Yes	All	Hernandez (2008)
SimuPop	Forward	CO	Yes	D, Pm, S	Nt: All	No	No	Yes	All	Peng and Kimmel (2005)

*“Recombination algorithm”: “SCR” means the standard coalescent with recombination to simulate the ARG (Hudson, 1983); “SMC” indicates the sequential Markovian coalescent, which is an approximation of the SCR (further details in, McVean and Cardin, 2005); “CO” means crossing over recombination model (see Padhukasahasram et al., 2008); “SB” indicates sex-biased recombination. “Other evolutionary processes”: “D,” “Pm,” and “S” mean demographics, population structure with migration, and selection, respectively. “Substitution model” refers to substitution models based on nucleotide “Nt” or codon “Cod” sequences; “Nt: All” means all nucleotide substitution models (JC, …, GTR). “Rate variation” indicates variable substitution rate across sites (G, gamma distribution; I, proportion of invariable sites). “Intracodon recombination” indicates if recombination breakpoints can occur at any codon position (Yes) or are forced to occur between codons (No). “OS” shows the availability of executable files in different operating systems (“All” means available for Macintosh, Windows, and Linux), “SC” means that the source code is available*.

*^a^The simulated ARG can be exported from *NetRecodon* and then can be visualized and analyzed using *NetTest* (Arenas et al., 2010)*.

*^b^Under codon models, dN/dS can vary across codons*.

*^c^Coding sequences are simulated by nucleotide substitution models, avoiding stop codons*.

### Simulation of coding DNA sequences with recombination

Direct simulation of coding DNA sequences with recombination can be only performed with a few programs. Using the coalescent approach, the programs *Recodon* (Arenas and Posada, 2007), *CodonRecSim* (Anisimova et al., 2003), and *NetRecodon* (Arenas and Posada, 2010a) allow such simulation, but only the latter program does not force recombination breakpoints to occur between codons, thus allowing more realistic simulations (see Arenas and Posada, 2010a). Concerning the forward-time approach, only the programs *GenomePop* (Carvajal-Rodriguez, 2008) and *SFS_CODE* (Hernandez, 2008) implement the simulation of coding sequences with recombination.

**Figure 1:**
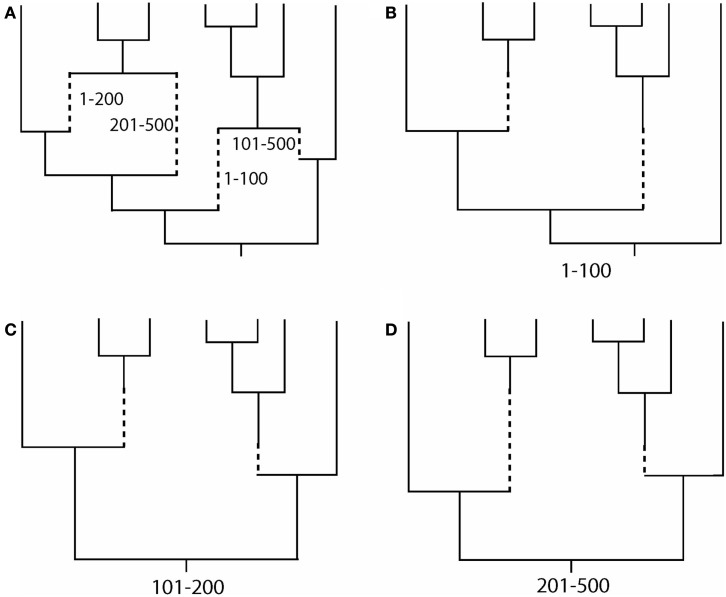
**Example of an ancestral recombination graph (ARG) with the corresponding embedded trees for each recombinant fragment**. **(A)** ARG based on two recombination events with breakpoints at positions 100 and 200. Dashed lines indicate branches for recombinant fragments. (**B–D**) Embedded tree for each recombinant fragment. Note that topologies and branch lengths may differ across trees. Finally, the simulation of sequence evolution can be performed site by site along the corresponding tree (see, Yang, 2006; Fletcher and Yang, 2009).

Evolutionary scenarios that are not implemented in these programs can be simulated by the following alternative methodology, which is based on the concatenation of two different simulators. First, we simulate an evolutionary history with recombination [an ancestral recombination graph (ARG, see Figure 1A), which contains a tree for each recombinant fragment; Figures 1B–D]. This procedure can be carried out using, for example, the program *ms* (Hudson, 2002); see also other evolutionary history simulators in (Hoban et al., 2012). Next, we simulate molecular evolution of each coding fragment, according to a user-specified codon-substitution model, along its corresponding simulated tree (further details in Yang, 2006; Fletcher and Yang, 2009). Finally, we just concatenate the simulated coding fragments. The simulation of coding sequence evolution along given trees can be performed, for example, with the program *INDELible* (Fletcher and Yang, 2009); see also other software in (Arenas, 2012; Arenas and Posada, 2012). The limitation of this methodology is that recombination breakpoints are always assumed to occur between codons and not within codons.

### Simulation of nucleotide sequences with recombination

A number of computer programs can directly simulate non-coding DNA sequences under recombination (see Table 1). Similarly to the previous subsection, the simulation of non-coding DNA sequences under other evolutionary scenarios, which are not described in the Table 1, can be performed by combining two computer tools. We can use a simulator of recombination evolutionary histories (e.g., *ms* or *msms*; Ewing and Hermisson, 2010) followed by a non-coding DNA sequence evolution simulator (e.g., *INDELible*, *Seq-Gen*, Rambaut and Grassly, 1997; *EVOLVER*, Yang, 1997; or *indel-Seq-Gen*, Strope et al., 2009).

### Simulation of genomes with recombination hotspots

It is known that the recombination rate is not homogeneous throughout the genome and some regions (hotspot regions) are more likely to suffer recombination (e.g., Gabriel et al., 2002; Zhuang et al., 2002). Consequently, recombination hotspots should be considered for realistic genome simulation.

The simulation of genomes with recombination requires robust and memory-efficient simulators. Programs like *fastsimcoal* (Excoffier and Foll, 2011) or *mlcoalsim* (Ramos-Onsins and Mitchell-Olds, 2007) allow for efficient simulations of non-coding genomic regions under recombination (including recombination hotspots). However, these tools do not implement a variety of substitution models (e.g., codon models), or particular evolutionary mechanisms like selection; this may be problematic if we are trying to mimic genome-wide data (see, Arbiza et al., 2011).

Again, an alternative methodology consists of the use of two simulators. A few programs currently implement the simulation of recombination hotspots, namely, *SNPsim* (Wiuf and Posada, 2003), *cosi* (Schaffner et al., 2005), *GENOME* (Liang et al., 2007), *mbs* (Teshima and Innan, 2009), and *msHOT* (Hellenthal and Stephens, 2007). Although all these programs simulate particular genetic markers (such as SNPs or STRs), DNA sequence evolution can be simulated upon phylogenetic trees produced by these programs if we use the two-step procedure described above.

### Simulation of recombination phylogenetic networks

In order to represent a full evolutionary history with recombination, phylogenetic networks should be used instead of forcing the genealogy onto a single tree (Huson and Bryant, 2006). There are two commonly used methodologies for the simulation of recombination networks: direct simulation of the ARG (e.g., Figure 1A) or combining the simulated trees for each recombinant fragment (e.g., Figures 1B–D). To my knowledge, only two programs can really output a simulated ARG, namely, *Serial NetEvolve* (Buendia and Narasimhan, 2006) and *NetRecodon* (Arenas and Posada, 2010a), where the ARG can be visualized and analyzed using the *NetTest* web server (Arenas et al., 2010)^1^. On the other hand, trees can be combined to generate a network using tools like *CombineTrees* (see for a review, Woolley et al., 2008)^2^.

## Recombination Simulation for Analyzing the Influence of Recombination on Phylogenetic Inferences

This section outlines three computer simulation studies where ignoring recombination leads to biased phylogenetic inferences. Alternative phylogenetic inference methodologies considering recombination are also suggested.

### Influence of recombination on phylogenetic tree reconstruction

Schierup and Hein (2000a) simulated samples under the coalescent with recombination (Hudson, 1983). Then, from the simulated genealogy, they simulated nucleotide sequence evolution under the Jukes-Cantor (JC) and Kimura’s two-parameter (K2P) nucleotide substitution models of evolution. The simulated datasets were analyzed using programs for phylogenetic tree reconstruction by both distance-based methods and maximum-likelihood (ML) methods. Ignoring recombination biased the inferred phylogenetic trees toward larger terminal branches, smaller times to the most recent common ancestor (MRCA) and incorrect topologies (Schierup and Hein, 2000a). In addition, ignoring recombination led to overestimation of the substitution rate heterogeneity, apparent homoplasies and loss of molecular clock (Schierup and Hein, 2000a,b). Later, Posada (2001) analyzed the molecular clock hypothesis on four empirical datasets. In particular, the author applied a triplet likelihood ratio test (test for equality of evolutionary rates among three species, called a relative-rate test, RRT), which is independent of topology and might be unbiased by recombination. Results showed that recombinant data did not allow a good fit to the molecular clock when using classical likelihood ratio tests (LRT). However, the molecular clock was not rejected when using the RRT test. Thus, this test could be recommended for testing a molecular clock in the presence of recombination. In addition, phylogenetic incongruence in empirical data was also observed as a consequence of ignoring recombination (e.g., Worobey and Holmes, 1999; Feil et al., 2001).

These findings, consequently, suggest biases in derived evolutionary analyses based on phylogenetic reconstructions that ignore recombination. As an alternative, there are two methodologies of phylogenetic reconstruction accounting for recombination:

– Inference of a single phylogenetic network (e.g., Figure 1A; Griffiths and Marjoram, 1997; Huson and Bryant, 2006). Recombination networks can be inferred by using computer programs like *SplitsTree* (Huson, 1998; Huson and Bryant, 2006).
– Inference of a set of phylogenetic trees, where each tree corresponds to the evolutionary history of each recombinant fragment (e.g., Figures 1B–D). The methodology consists of the detection of recombination breakpoints (see for a review, Martin et al., 2011) followed by a phylogenetic tree reconstruction for each recombinant fragment.

Both methodologies correctly account for recombination and the choice should be based on the posterior application. For example, the phylogenetic network may help for an easy visualization of clades and phylogenetic relationships (e.g., Maughan and Redfield, 2009). By contrast, the simulation of sequence evolution requires a phylogenetic tree for each recombinant fragment (e.g., Fletcher and Yang, 2009).

### Influence of recombination on ancestral sequence reconstruction

Recently, Arenas and Posada (2010c) analyzed the effect of considering recombination on ancestral sequence reconstruction (ASR). They performed extensive simulations of nucleotide, codon, and amino acid data by using the coalescent with recombination approach implemented in *NetRecodon*. They then reconstructed ancestral sequences with different ASR methods (joint ML, marginal ML, and empirical Bayes). Results clearly indicated that ignoring recombination biases the reconstruction of ancestral sequences, regardless of the method or software used. This ASR error can be partially reduced if recombination is considered (Arenas and Posada, 2010c). The methodology consists of four steps: the detection of recombination breakpoints, the reconstruction of a phylogenetic tree for each recombinant fragment, the reconstruction of ancestral fragments by using the corresponding trees and, finally, the concatenation of the ancestral fragments to generate the entire ancestral sequence. The *Datamonkey* web server (Kosakovsky Pond and Frost, 2005)^3^ and the *Hyphy* package (Kosakovsky Pond et al., 2005) have automated the whole procedure described above to infer ancestral sequences with consideration of recombination.

Arenas and Posada (2010c) also analyzed empirical data, in particular two datasets of the *env* region of HIV-1. They inferred ancestral sequences both ignoring and considering recombination, using the methodology described above, and observed a different number of CTL epitopes depending on whether recombination was considered or not.

### Influence of recombination on the detection of molecular adaptation

The detection of molecular adaptation (based on the non-synonymous/synonymous substitution rate ratio, hereafter dN/dS) is commonly used at both global (entire sequence) and local (codon) levels. Indeed, these analyses have commonly been applied to datasets collected from highly recombinant viruses and bacteria (e.g., Perez-Losada et al., 2009, 2011; Bozek and Lengauer, 2010). Several studies have shown the impact of recombination on the estimation of dN/dS (e.g., Anisimova et al., 2003; Arenas and Posada, 2010a). After simulating coding data under a variety of codon-substitution models for heterogeneous selection pressure (see, Yang et al., 2000) and different levels of recombination, they selected those heterogeneous codon models that best fitted the simulated data by using LRTs. Results showed a weak impact of recombination on the estimation of global dN/dS but a strong effect on the estimation of local dN/dS, in particular by increasing the number of false-positively selected sites (PSS). An alternative methodology to reduce these errors consists of the detection of recombination breakpoints followed by the reconstruction of a phylogenetic tree for each recombinant fragment and, finally, the estimation of dN/dS by using the corresponding trees (see, Kosakovsky Pond et al., 2006). This methodology was applied in (Perez-Losada et al., 2009, 2011). Again, the *Datamonkey* web server and the *Hyphy* package have automated this whole procedure to estimate dN/dS while accounting for recombination.

Recombination might also affect other evolutionary inferences. For example, it could bias those analytical methods based on the coalescent without recombination (e.g., BEAST; Drummond and Rambaut, 2007). However these influences have not yet been rigorously evaluated.

Another interesting question concerns the influence of recombination on genetic diversity. Spencer et al. (2006) studied this in humans and found that recombination only affects genetic diversity at recombination hotspots. However, such hotspots did not alter substitution rates, perhaps because recombination rates were always low. By contrast, large recombination rates (common in a variety of viruses and bacteria) may strongly increase genetic diversity and bring novel lineages (e.g., He et al., 2010).

At this point, I would suggest the approximate Bayesian computation (ABC) approach (see for a review, Beaumont, 2010) to estimate evolutionary parameters accounting for recombination. ABC is based on computer simulations and provides an alternative for those analyses where the likelihood function cannot be computed. Simulations can be performed according to a prior distribution for recombination rate (among other parameters) and then, by a rejection or a regression method, a posterior distribution can be computed to obtain the parameter estimates (Beaumont et al., 2002). For example, Wilson et al. (2009) applied ABC for joint estimation of a set of evolutionary parameters, such as substitution rate, dN/dS and recombination rate. By this methodology, the influence of recombination on other evolutionary mechanisms is accounted for, but only if it is indeed implemented in the computer simulator.

## Conclusion

This review provides a practical guide to the state of the art in software, and recommends methodologies, for simulating coding and non-coding sequence data with recombination, including recombination hotspots. Currently, only three programs implement the direct simulation of coding data with recombination. These programs will not cover every evolutionary scenario, but this problem can be circumvented by the use of two simulators, one for the evolutionary history and another for sequence evolution. It is also important to consider intracodon recombination (Arenas and Posada, 2010a), because 2/3 of recombination events are expected to occur within codons. By contrast, the simulation of non-coding sequences with recombination can be performed by a variety of programs. Here again, two simulators may be combined where necessary.

Among many other applications (e.g., Sun et al., 2011; Marttinen et al., 2012), the simulation of DNA data with recombination has been especially important for demonstrating the strong influence of recombination on phylogenetic tree reconstruction and derived analyses, such as ASR or dN/dS estimation. However, some alternative methodologies have been developed for phylogenetic inference accounting for recombination.

The current set of computer tools to simulate DNA sequences with recombination can cover a wide range of evolutionary scenarios. However, some scenarios are still difficult to simulate and will require the development of more complex simulators. For example, next-generation sequencing (NGS) technologies now deliver fast and accurate genome sequences (Metzker, 2010) that may call for simulations of entire genomes accounting for recombination (including recombination hotspots; e.g., Westesson and Holmes, 2009; Marttinen et al., 2012), as well as other evolutionary mechanisms like natural selection. Indeed, the simulation of DNA evolution should be performed by using different substitution models for each genomic region (Arbiza et al., 2011). Moreover, I would expect interactions between the different evolutionary forces, such as joint influences of natural selection and recombination on dN/dS (e.g., Anisimova et al., 2003; Kryazhimskiy and Plotkin, 2008) or of structural protein energies on sequence evolution (e.g., Bastolla et al., 2007; Arenas et al., 2009; Grahnen et al., 2011). To my knowledge, there is currently no tool to simulate sequences accounting for all these evolutionary features, including interactions among them. On the other hand, there is also a demand for fast simulations, in particular for applying ABC or Bayesian model-choice approaches that require extensive simulations (see recombination examples in, Wilson et al., 2009; Nunes and Balding, 2010; Sohn et al., 2012).

In conclusion, there is a need to innovate continuously in fast and complex simulators of DNA sequences with recombination and I expect future advances in this area.

## Conflict of Interest Statement

The author declares that the research was conducted in the absence of any commercial or financial relationships that could be construed as a potential conflict of interest.

## Footnotes

^1^http://darwin.uvigo.es/software/nettest/

^2^http://applications.lanevol.org/combineTrees/

^3^http://www.datamonkey.org/
